# Elevated Level of Cerebrospinal Fluid Pyruvate Dehydrogenase Kinase 4 Is a Predictive Biomarker of Clinical Outcome after Subarachnoid Hemorrhage

**DOI:** 10.3390/brainsci12111507

**Published:** 2022-11-06

**Authors:** Xuan Gao, Huasheng Zhang, Zheng Peng, Zong Zhuang, Wei Li

**Affiliations:** 1 Department of Neurosurgery, Tianjin Huanhu Hospital, Tianjin 300333, China; 2 Department of Neurosurgery, Nanjing Drum Tower Hospital, The Affiliated Hospital of Nanjing University Medical School, Nanjing 210008, China

**Keywords:** subarachnoid hemorrhage, cerebrospinal fluid, pyruvate dehydrogenase kinase 4, prediction of recovery

## Abstract

Subarachnoid hemorrhage (SAH) is a central nervous system disease with high mortality and morbidity. Some independent factors valuable for prognosis prediction in patients with SAH are still lacking. In our earlier study, we found that PDK4 exerts a protective effect after SAH, primarily by reducing oxidative stress and neuronal death via the ROS/ASK1/p38 signaling pathway. Therefore, we investigated the changes in the level of pyruvate dehydrogenase kinase 4 (PDK4) in patients after subarachnoid hemorrhage (SAH) and analyzed the value of the cerebrospinal fluid (CSF) PDK4 level in predicting the prognoses of patients with SAH after interventional embolization surgery. Some knee arthritis subjects who needed surgery were recruited as a control group. The results showed that PDK4 expression was elevated in the CSF of SAH patients compared with that of controls. PDK4 levels in CSF (OR = 4.525; 95% CI: 1.135–18.038; *p* = 0.032), time to surgery (OR = 0.795; 95% CI: 0.646–0.977; *p* = 0.029), and initial GCS scores (OR = 2.758; 95% CI: 0.177–43.106; *p* = 0.469) were independent prognostic risk factors for SAH patients after surgery. The receiver operating characteristic (ROC) curve showed PDK4 levels in CSF had a higher predictive value. Thus, PDK4 in CSF could be an independent prognostic risk factor for SAH patients after surgery. PDK4 has the potential to serve as a new therapeutic target and biomarker for use in the diagnosis of SAH severity and the prediction of recovery.

## 1. Introduction

Subarachnoid hemorrhage (SAH), especially ruptured hemorrhage associated with aneurysms, is a severe disease of the central nervous system (CNS). According to the World Health Organization, the morbidity of subarachnoid hemorrhage is approximately 22.5 per 100,000 people globally [1], with a mortality rate of 67% [2]. Most survivors are known to suffer from severe neurological sequelae [3]. In the past, cerebral vasospasm was considered the leading cause of poor prognosis in patients with delayed cerebral ischemia; however, research has confirmed that cerebral vasospasm is not responsible for patient prognosis [4,5]. Moreover, independent factors valuable for prognosis prediction in patients with SAH are still lacking. Currently, the Glasgow Coma Scale (GCS), Modified Fisher Scale, World Federation of Neurological Surgeons grade (WFNS), and Hunt and Hess score all depend on the patient’s description and subjective judgment, which are not completely objective evaluation factors [6,7,8]. Thus, it is essential to investigate and discover targets that are completely objective, immediate, effective, and independently predicting patient outcomes.

Early brain injury is another factor in the poor prognosis of SAH patients, which promotes the development of delayed cerebral ischemia [9]. Early brain injury refers to pathological changes occurring within 72 h of SAH, mainly including cell death, oxidative stress, mitochondrial damage, metabolic disorders, and other pathological conditions [10,11]. Detecting the severity of early brain injury in SAH patients may be helpful in predicting their prognosis.

Recently, several studies have shown that neuronal energy metabolism disorders, including changes in the ATP supply, intracellular calcium concentration, reduction-oxidation potential, and free radical scavenging, are common events in most brain injuries. Pyruvate dehydrogenase kinase 4 (PDK4), a key enzyme located on the outer mitochondrial membrane and involved in glycolysis and the tricarboxylic acid cycle (TCA), directly affects the energy metabolism status of cells [12,13,14]. PDK4 acts as a regulator of mitochondrial metabolism and can inactivate pyruvate dehydrogenase (PDH) by phosphorylation. Inactivated PDH affects the conversion of pyruvate to the TCA [15]. Thus, the expression level of PDK4 could directly reflect the state of neuronal metabolism. In our earlier study, we found that, among the four isozymes of PDK, only PDK4 expression was significantly increased in the brain after SAH. PDK4 exerts a protective effect after SAH, primarily by reducing oxidative stress and neuronal death via the ROS/ASK1/p38 signaling pathway [16]. However, whether PDK4 plays the same role in SAH patients as in animals remains unknown. Nevertheless, the results of the present study suggested that PDK4 may serve as a new therapeutic target and biomarker for the diagnosis of SAH severity and the prediction of recovery. Therefore, we collected cerebrospinal fluid (CSF) samples from patients with SAH and detected the contents of PDK4 and related metabolites. We further analyzed whether PDK4 could be an independent prognostic risk factor for SAH patients after surgery.

## 2. Materials and Methods

### 2.1. Patient Population

Patients with subarachnoid hemorrhage admitted to the Department of Neurosurgery, Nanjing Drum Tower Hospital, the Affiliated Hospital of Nanjing University Medical School, from April 2020 to April 2021 were selected for the study. For all SAH subjects, the inclusion criteria were as follows:

(1) According to the Chinese Guidelines for the Diagnosis and Treatment of Subarachnoid Hemorrhage 2019 [17], all patients were diagnosed by brain CT, CT angiography (CTA) or whole cerebral angiography (DSA), and lumbar puncture;
(2) The first onset, the age of onset, is 16–70 years old (including the critical value);
(3) Signed consent from the subject or next of kin.

The exclusion criteria were as follows:

(1) Failure to meet the inclusion criteria or being unfit for the experiment as determined by the responsible doctor;
(2) Presence of severe cardiac insufficiency, renal dysfunction, diabetes, or other systemic diseases;
(3) A history of traumatic brain injury, severe cerebral edema, or hydrocephaly.

As a control group, subjects with knee arthritis who needed surgery were recruited. For control subjects, the inclusion criteria were as follows:

(1) Those who had been diagnosed with gonarthritis by a professional doctor requiring spinal anesthesia;
(2) Patients aged 16–70 years (including cutoff values);
(3) No current or pre-existing brain injuries, neurological diseases, or bleeding disorders, and subjects who needed surgery for knee arthritis;
(4) Signed consent from the subject or next of kin.

Ultimately, 34 patients with subarachnoid hemorrhage and six controls were enrolled, and the basic information of the patients is presented in Table 1. This study was approved by the Ethics Committee of Nanjing Drum Tower Hospital, the Affiliated Hospital of Nanjing University Medical School (Approval No. 2020-041-01) and registered in the China Clinical Trial Registry (Registration No. Chi CTR 2100042986).

### 2.2. Sample Collection

All cerebrospinal fluid samples were collected in accordance with the consensus protocol for the standardization of cerebrospinal fluid collection. Cerebrospinal fluid samples were collected from patients with subarachnoid hemorrhage during postoperative lumbar puncture to check the cerebrospinal fluid of the patients and assess their condition. Cerebrospinal fluid samples were collected from patients in the control group during preoperative spinal anesthesia. The posterior cerebrospinal fluid was retained as a sample and centrifuged at 2000× *g* for 15 min at 4 °C to pellet cellular bodies and debris. All samples were stored at −80 °C.

### 2.3. Enzyme-Linked Immunosorbent Assay (ELISA)

Levels of PDK4 in cerebrospinal fluid were detected using an ELISA kit (abx252933, Abbexa). The prepared cerebrospinal fluid samples and provided standards were added to 96 empty plates with PDK4-specific antibody adsorbed, and the reaction was stopped by adding reaction solution, which was detected at OD 450 nm on a microplate reader. The PDK4 content in each sample was calculated from a standard curve.

### 2.4. Pyruvate Assay

Levels of pyruvate in cerebrospinal fluid were determined using a dedicated pyruvate assay (# K609-100, BioVision, Palo Alto, CA, USA). Cerebrospinal fluid samples and standards were added to 96-well plates according to the instructions, and reaction solutions were added and finally detected under the conditions of the microplate reader set at OD 570 nm to calculate the pyruvate level in the samples according to the standard curve.

### 2.5. Data Collection and Analysis

Patients included in this study were recruited based on sex, age, previous history of hypertension, presence of a responsible aneurysm on DSA examination, time from onset to surgery, number of leukocytes, PDK4, and pyruvate in CSF, and time of CSF collection after onset. Other parameters, such as the GCS score assessed at admission, the Hunt-Hess grade, the Glasgow Outcome Scale (GOS) score at discharge, and the GOS score three months after discharge, were also considered. The patients were divided into the good prognosis group (GOS = 5 points) and the poor prognosis group (GOS < 5 points) according to the GOS score three months after discharge. Univariate and multivariate logistic regression analyses were done to determine the differences in clinical data and biochemical parameters between the two groups and the independent factors predicting the prognosis of patients with SAH.

### 2.6. Statistical Analysis

Statistical analysis was performed using SPSS 19.0 software. Clinical data were described as percentages, medians, and quartiles. The Mann–Whitney test and chi-square test were used to compare whether there were any differences between the two groups. A univariate regression analysis was performed to explore possible factors associated with the prognosis of SAH patients. Multivariate logistic regression analysis was performed to verify and compare the effectiveness of each independent factor. The ROC curve visually shows how each independent factor predicts patient outcome. *p* < 0.05 was considered statistically significant.

## 3. Results

### 3.1. Patient Characteristics and an Overview of Upgrading

We collected 40 CSF samples: six from normal patients (control) and 34 from SAH patients. Baseline characteristics are displayed in Table 1. There was no significant difference in the age distribution (*p* = 0.544) or gender composition (*p* = 0.792) between the control and SAH groups. The levels of PDK4 in CSF were significantly increased in SAH patients compared with controls (7.2 [IQR: 5.6–8.6] vs. 3.5 [IQR: 3.3–3.9] ng/mL, *p* = 0.003). Pyruvate content was also markedly increased (11.6 [IQR: 8.6–11.9] vs. 3.9 [IQR: 1.9–4.4] mM, *p* = 0.001). Around 20 patients in the SAH group had a previous history of hypertension, 29 had significant aneurysms on preoperative cerebral angiography after onset, and the time from onset to surgical treatment was approximately 18.8 h [IQR: 12.0–24.0]. CSF samples were taken on day 1 (8 patients), day 2 (14 patients), and day 3 (12 patients) after surgery. There were no differences in GCS scores or Hunt-Hess scores between the groups. CSF samples from SAH patients showed the presence of white blood cell (WBC) counts of 177.0 [IQR: 7 2.0–351.0] × 10^6^ cells/L and red blood cell (RBC) counts of 83,500.0 [IQR: 29,750.0–209,750.0] × 10^6^ cells/L. SAH patients were stratified into those with good or poor outcomes based on GOS. At discharge, 15 patients (44.1%) had a good outcome (GOS = 5), while 19 (55.9%) of them had a poor outcome (GOS < 5). After three months, 26 patients (76.5%) had a good outcome, while eight patients (23.5%) had a poor outcome.

### 3.2. Clinical Data and Comparison of Patients in the Good and Poor Outcome Groups

No significant difference was observed in the age distribution (*p* = 0.935) or gender (*p* = 0.961) between the good and poor outcome groups. There was no difference (*p* = 0.161) between the two groups with respect to the previous history of hypertension (*p* = 0.840). All the patients had significant responsible aneurysms (84.6% vs. 87.5%, *p* = 0.840), and the timing of CSF sampling had no significant impact on the outcome (*p* = 0.664). Patients with a good outcome had a shorter time from onset to surgery than those with a poor outcome (18.3 h [IQR: 12.0–23.3] vs. 20.0 [IQR: 14.3–24.5], *p* = 0.046). The GCS score at admission was significantly higher in the good outcome group than in the poor outcome group (*p* = 0.008), and there was no significant difference in Hunt-Hess grade distribution between the good outcome group and the poor outcome group (*p* = 0.317). Further, we found that there was no significant difference in the number of WBCs in the CSF (182.5 [IQR: 76.5–416.0] vs. 131.5 [IQR: 25.3–313.3] × 10^6^ cells/L, *p* = 0.118) and a little change in the number of RBCs (83,500.0 [33,250.0–246,250.0] vs. 65,000.00 [25,250.00–155,250.00] × 10^6^ cells/L, *p* = 0.761) in the poor outcome group compared to the good outcome group. PDK4 content was significantly higher (7.7 [6.2–8.8] vs. 5.2 [4.1–6.7] ng/mL, *p* = 0.002), as was pyruvate content (11.7 [11.4–12.5] vs. 7.9 [6.3–8.2] mM, *p* = 0.000). The GOS score at discharge was not significantly different from that at three months’ follow-up after discharge (*p* = 0.213).

### 3.3. Univariate and Multivariate Regression Analysis for Predicting Postoperative Outcome

**Figure 1:**
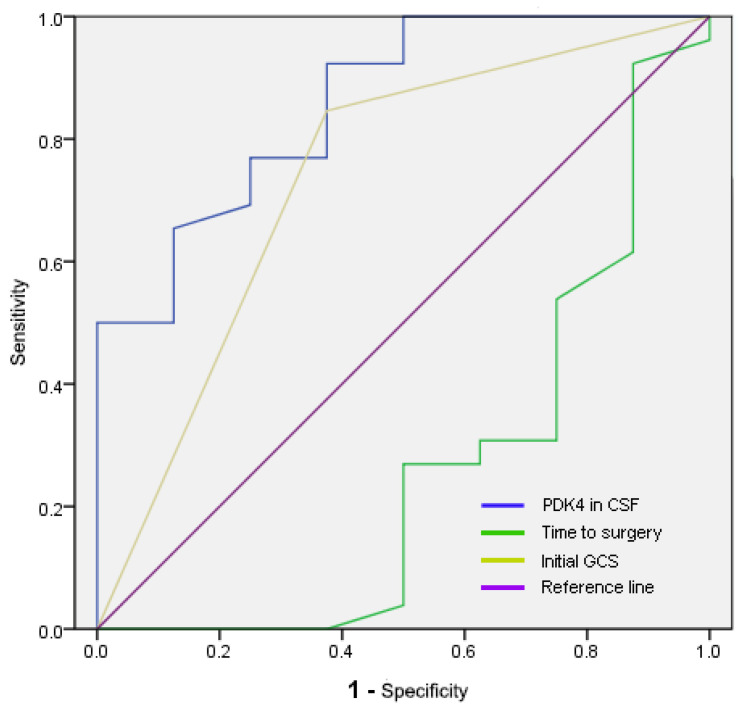
ROC analysis to compare the diagnostic accuracy of the identified parameters. PDK4: pyruvate dehydrogenase kinase 4, GCS: Glasgow Coma Scale.

As shown in Table 2, a univariate analysis of the prediction of postoperative outcome showed that PDK4 content in CSF, the time interval from onset to surgery, and the initial GCS score were closely related to the prognosis of patients. Although a link was observed between the GOS score at the time of the patient’s initial discharge and their outcome, the nonparametric tests showed no difference between them and the patient’s prognosis at 3 months. Therefore, it was considered a false positive. In multivariate regression analysis, PDK4 content in CSF (OR = 4.525; 95% CI: 1.135–18.038; *p* = 0.032) and the time interval from onset to surgery (OR = 0.795; 95% CI: 0.646–0.977; *p* = 0.029) had independent predictive values for postoperative outcome in patients. The predictive value parameters of these independent factors were visually demonstrated using ROC analysis in Figure 1 and Table 3. The ROC analysis provided the subthreshold area, sensitivity, specificity, and cut-off value of each independent predictor, reflecting the independent predictive value of each factor in more detail and accuracy.

## 4. Discussion

Our study is the first to show that PDK4 content in CSF was significantly increased in SAH patients compared with controls, while pyruvate levels were also significantly increased. This reflected the increased expression of PDK4 after SAH, changed the metabolic status of neurons, and truncated the path of pyruvate into the TCA. In many studies of hypermetabolism-related diseases such as diabetes and liver injury, PDK4 was found to reduce oxidative stress and play a cytoprotective role [18,19]. PDK4 has also been investigated in the occurrence, development, and migration of cancer. Moreover, high expression of PDK4 has been found to accelerate the growth of cancer cells and promote metastasis, such as in bladder, breast, and intestinal cancer [20,21,22]. Accumulated pyruvate resulting from increased expression of PDK4 promotes the clearance of reactive oxygen species, reduces oxidative stress, and plays a cytoprotective role, contributing to the white matter remodeling following traumatic brain injury [23,24]. Therefore, it is worth exploring the correlation between the changes in PDK4 and pyruvate levels in the CSF of SAH patients and the prognosis.

After analyzing the data of SAH patients, we confirmed that PDK4 content in CSF had an independent predictive value for the postoperative outcome. The higher the PDK4 content, the better the prognosis for patients. It was also found that the time interval from onset to surgery also had independent predictive value for the postoperative outcome, but the longer the interval, the worse the prognosis for patients. Other clinical factors did not have independent predictive value for the postoperative outcome. This was consistent with the results of a large clinical study abroad. Frank et al. analyzed the treatment and prognosis of more than 90,000 patients throughout the United States. They concluded that patients with delayed treatment were associated with an increased rate of moderate to severe neurological disability [25]. PDK4, as a key enzyme involved in glycolysis and the TCA on the outer mitochondrial membrane, directly affects the energy metabolism status of cells [12,13,14]. Metabolic changes in the brain have been thought to be closely associated with brain injury. Diene and Cruz et al. suggested that increased accumulation of lactate in the CSF may be associated with brain injury. Some scientists have proposed lactate levels in the CSF as a prognostic predictor in SAH patients, but it is still controversial [26,27,28,29]. Lactic acid accumulation following brain injury arises from increased PDK4. Increased PDK4 could promote the phosphorylation of PDH and inhibit PDH activity. It prevents pyruvate from entering the TCA and thus leads to pyruvate accumulation. Hence, pyruvate enters other metabolic pathways, and generates lactic acid, thus releasing energy [30,31]. Therefore, although there was a significant difference in pyruvate content in CSF between the good and poor outcome groups, univariate regression analysis showed that the change in pyruvate content was not associated with prognostic changes. This also suggests that lactate is not suitable as a SAH prognostic predictor. PDK4 is the cumulative initiation point of pyruvate and lactate accumulation after brain injury. Our results showed that PDK4 levels in CSF have an independent predictive value for the patient’s postoperative outcome. Analysis of the predictive value of each independent predictor in Figure 1 and Table 3 showed that the sensitivity and specificity of PDK4 content in CSF were higher with respect to all three factors. The higher the PDK4 content, the better the patient’s prognosis. PKD4 has the potential to be a new therapeutic target and a prognostic and predictive target in SAH patients.

The number of patients in the poor prognosis group was small among the first enrolled patients, and they had mainly mild SAH. In CNS injury, the targets detected from CSF can better reflect the damage to brain parenchyma. Most patients with mild SAH need to undergo lumbar puncture and drainage of part of the CSF for detection and treatment. Therefore, the immediate lumbar puncture in SAH patients may be helpful, and the biomarkers detected in the CSF may meet the requirements for independently predicting the prognosis targets of patients [32]. Many patients with severe SAH have poor conditions, and lumbar puncture may lead to a brain hernia and affect the treatment of the patients. Therefore, the postoperative outcome of patients with mild SAH is mainly studied. Although our study highlights the critical details of patient prognosis, the limitations mentioned above warrant further studies.

## 5. Conclusions

PDK4 expression was elevated in the CSF of SAH patients compared with that of controls. PDK4 in CSF could be an independent prognostic risk factor for SAH patients after surgery. PDK4 has the potential to serve as a new therapeutic target and biomarker for use in the diagnosis of SAH severity and the prediction of recovery.

## Figures and Tables

**Table 1:** Baseline characteristics of control and SAH patients stratified by outcome.

Items	Control	SAH (n = 34)				
		All	*p* Value	Good Outcome	Poor Outcome	*p* Value
Participants (n)	6	34	-	26	8	-
Age (years)	56.5 (42.5–66.0)	59.5 (51.5–66.5)	0.544	59.5 (51.5–66.5)	59.5 (49.75–66.5)	0.935
Gender			0.792			0.961
male	3 (50%)	13 (38.2%)		10 (38.5%)	3 (37.5%)	
female	3 (50%)	21 (61.8%)		16 (61.5%)	5 (62.5%)	
Hypertension			-			0.161
<140/90	-	14 (41.2%)		9 (34.6%)	5 (62.5%)	
≥140/90	-	20 (58.8%)		17 (65.4%)	3 (37.5%)	
Aneurysm			-			0.840
No	-	5 (14.7%)		4 (15.4%)	1 (12.5%)	
Yes	-	29 (85.3%)		22 (84.6%)	7 (87.5%)	
Time to surgery (h)	-	18.8 (12.0–24.0)	-	18.3 (12.0–23.3)	20.0 (14.3–24.5)	**0.046**
Initial GCS			-			**0.008**
>12	-	25 (73.5%)		22 (84.6%)	3 (37.5%)	
≤12	-	9 (26.5%)		4 (15.4%)	5 (62.5%)	
Initial Hunt-Hess			-			0.317
3–4	-	16 (47.1%)		11 (42.3%)	5 (62.5%)	
1–2	-	18 (52.9%)		15 (57.7%)	3 (37.5%)	
CSF (days, after hemorrhage)			-			0.664
1	-	8 (23.5%)		5 (19.2%)	3 (37.5%)	
2	-	14 (41.2%)		12 (46.2%)	2 (25%)	
3	-	12 (35.3%)		9 (34.6%)	3 (37.5%)	
White cells in CSF (×10^6^)	-	177.0 (72.0–351.0)	-	182.5 (76.5–416.0)	131.5 (25.3–313.3)	0.118
Red cells in CSF (×10^6^)	-	83500.0 (29750.0–204000.0)	-	83500.0 (33250.0–246250.0)	65000.00 (25250.00–155000.00)	0.761
PDK4 in CSF (ng/mL)	3.5 (3.3–3.9)	7.2 (5.6–8.6)	**0.003**	7.7 (6.2–8.8)	5.2 (4.1–6.7)	**0.002**
Pyruvate in CSF (mM)	3.9 (1.9–4.4)	11.6 (8.6–11.9)	**0.001**	11.7 (11.4–12.5)	7.9 (6.3–8.2)	**0.000**
GOS (after surgery)			-			0.213
5	-	15 (44.1%)		13 (50%)	2 (25%)	
<5	-	19 (55.9%)		13 (50%)	6 (75%)	

GCS: Glasgow Coma Scale, GOS: Glasgow Outcome Scale, PDK4: pyruvate dehydrogenase kinase 4, SAH: subarachnoid hemorrhage.

**Table 2:** Univariable and multivariable logistic regression analyses of possible predictors for the prognosis of SAH patients.

Parameters	Univariable Logistic Regression			Multivariable Logistic Regression		
	OR	95% CI	*p* Value	OR	95% CI	*p* Value
Age (years)	0.994	0.915–1.081	0.894			
Gender (male/female)	1.042	0.203–5.343	0.961			
Hypertension	3.148	0.608–16.289	0.171			
<140/90						
≥140/90						
Aneurysm	0.786	0.075–8.243	0.841			
No						
Yes						
Time to surgery (h)	0.876	0.773–0.992	**0.037**	0.795	0.646–0.977	**0.029**
Initial GCS	9.167	1.539–54.592	**0.015**	2.758	0.177–43.106	0.469
>12						
≤12						
Initial Hunt-Hess	0.44	0.086–2.244	0.323			
3–4						
1–2						
CSF (days, after hemorrhage)	1.306	0.459–3.713	0.617			
1						
2						
3						
White cells in CSF (×10^6^)	1.003	0.998–1.008	0.237			
Red cells in CSF (×10^6^)	1	1.000–1.000	0.507			
PDK4 in CSF (ng/mL)	2.998	1.254–7.166	**0.014**	4.525	1.135–18.038	**0.032**
Pyruvate in CSF (mM)	11462	0.000–1.384E12	0.325			
GOS (after surgery)	3.296	0.508–17.708	**0.035**			

GCS: Glasgow Coma Scale, GOS: Glasgow Outcome Scale, PDK4: pyruvate dehydrogenase kinase 4, SAH: subarachnoid hemorrhage.

**Table 3:** Diagnostic accuracy for the prediction of the prognosis of SAH patients.

Parameters	AUC (95% CI)	Cut-off	Sensitivity	Specificity
PDK4 in CSF (ng/mL)	0.858 (0.714–1)	5.54183	0.923	0.625
Time to surgery (h)	0.264 (0.03–0.499)	6.75	0.923	0.125
Initial GCS	0.736 (0.517–0.954)	0.6	0.846	0.625

GCS: Glasgow Coma Scale, PDK4: pyruvate dehydrogenase kinase 4.

## Data Availability

The datasets supporting the conclusions of this article are included within the article.
